# Phylogenetic diversity and activity screening of cultivable Actinobacteria isolated from marine sponges and associated environments from the western coast of India

**DOI:** 10.1099/acmi.0.000242

**Published:** 2021-09-21

**Authors:** Ulfat Baig, Neelesh Dahanukar, Neha Shintre, Ketki Holkar, Anagha Pund, Uttara Lele, Tejal Gujarathi, Kajal Patel, Avantika Jakati, Ruby Singh, Harshada Vidwans, Vaijayanti Tamhane, Neelima Deshpande, Milind Watve

**Affiliations:** ^1^​ Indian Institute of Science Education and Research, Pune (IISER-P), Dr Homi Bhabha Road, Pashan, Pune 411008, Maharashtra, India; ^2^​ Department of Microbiology, M.E.S. Abasaheb Garware College, Pune 411004, Maharashtra, India; ^3^​ Institute of Bioinformatics and Biotechnology, Savitribai Phule Pune University, Pune 411007, Maharashtra, India; ^4^​ Behavioural Intervention for Lifestyle Disorders (BILD) Clinic, Deenanath Mangeshkar Hospital and Research Centre, Erandwane, Pune 411004, Maharashtra, India

**Keywords:** bacterial predation, antibiotic production, secondary metabolites, enzyme inhibition, molecular phylogeny

## Abstract

The phylogenetic diversity of cultivable actinobacteria isolated from sponges (*Haliclona* spp.) and associated intertidal zone environments along the northern parts of the western coast of India were studied using 16S rRNA gene sequences. A subset of randomly selected actinobacterial cultures were screened for three activities, namely predatory behaviour, antibacterial activity and enzyme inhibition. We recovered 237 isolates from the phylum Actinobacteria belonging to 19 families and 28 genera, which could be attributed to 95 putative species using maximum-likelihood partition and 100 putative species using Bayesian partition in Poisson tree processes. Although the trends in the discovery of actinobacterial genera isolated from sponges were consistent with previous studies from different study areas, we provide the first report of nine actinobacterial species from sponges. We observed widespread non-obligate epibiotic predatory behaviour in eight actinobacterial genera and we provide the first report of predatory activity in *Brevibacterium *, *Glutamicibacter *, *Micromonospora *, *Nocardiopsis *, *Rhodococcus* and *Rothia *. Sponge-associated actinobacteria showed significantly more predatory behaviour than environmental isolates. While antibacterial activity by actinobacterial isolates mainly affected Gram-positive target bacteria with little or no effect on Gram-negative bacteria, predation targeted both Gram-positive and Gram-negative prey with equal propensity. Actinobacterial isolates from both sponges and associated environments produced inhibitors of serine proteases and angiotensin-converting enzyme. Predatory behaviour was strongly associated with inhibition of trypsin and chymotrypsin. Our study suggests that the sponges and associated environments of the western coast of India are rich in actinobacterial diversity, with widespread predatory activity, antibacterial activity and production of enzyme inhibitors. Understanding the diversity and associations among various actinobacterial activities – with each other and the source of isolation – can provide new insights into marine microbial ecology and provide opportunities to isolate novel therapeutic agents.

## Introduction

The marine ecosystem is not only diverse with respect to the micro-organisms found in it but also the natural products being synthesized by these micro-organisms [1–3]. Actinobacteria are among the taxa rich in secondary metabolites [4] and are widely distributed in diverse habitats, including soil, marine and freshwater and sediment [1, 2, 5–8]. They are also common in extreme environments [9–14] and are found as endobiotic symbionts of higher organisms [2, 15–19]. They belong to the phylum Actinobacteria and represent one of the major phyla within the bacterial domain [20]. Widely distributed in aquatic and terrestrial habitats, actinobacteria are Gram-positive or Gram-variable aerobes, facultative anaerobes or anaerobes with rigid cell wall containing muramic acid, and are phenotypically diverse, ranging from cocci to highly differentiated mycelia [20]. Marine ecosystems are believed to have a wide range of unexplored actinobacteria [21] and their metabolites [2, 3, 22], with diverse biological activities such as anticancer [23], anti-inflammatory [24], antibiotic [25–27], cytotoxic [28] and enzyme inhibitory [29, 30] activity. Watve *et al.* [31] estimated that the genus *Streptomyces* alone is capable of producing up to 10^5^ different metabolites, the majority of which remain unexplored. Of 23 000 medicinally important metabolites produced by marine micro-organisms, 70 % are contributed by actinobacteria [32]. To date, 8 Actinobacteria genera have been reported to produce secondary metabolites and 267 products have been reported from 96 marine actinobacteria [33].

Ecologically, it is difficult to understand the production of extracellular metabolites or enzymes by aquatic bacteria, since any molecule secreted outside the cell can be quickly washed off [34, 35]. Extracellular products could be useful to the producer only in viscous or partially enclosed environments. In the marine environment, sponges are likely to provide such a closed environment for bacteria. Sponges are filter feeders and collect small nutrient particles, including bacteria. This makes the environment locally nutrient-rich in otherwise oligotrophic surroundings. Bacteria, especially actinobacteria, isolated from these sponges may live in a symbiotic relationship that helps the host with defence against predation, sponge skeleton stabilization, translocation of metabolites and the nutritional process [2, 17, 21, 25–27, 36, 37]. In addition, since sponges are sessile and lack other anti-predator defences, secondary metabolites of bacteria can provide them with chemical defence [38]. Therefore, we expect more secondary metabolite related activities from sponge-associated actinobacteria.

Sponge-associated actinobacteria are likely to have another ecological role. In the phylum Actinobacteria, at least three genera, namely *Agromyces *, *Streptomyces* and *Streptoverticillium *, have been shown to be predators that kill and feed on other live bacterial cells [39–42]. Kumbhar and Watve [34] argued that antibiotic activity might have evolved primarily as a weapon in predation. However, the expression of secondary metabolites during predation may be independent of antibiotic expression in pure culture; the latter is likely to have evolved for mutualism with higher animal or plant hosts [43, 44]. We hypothesize that, for a niche of predation in association with sponge, the predatory actinobacterial species need to protect themselves from the digestive enzymes of the sponge as well as their own enzymes used for predation. Therefore, predatory actinobacteria are also expected to have efficient inhibitors of lytic enzymes.

The northern parts of the western coast of India, along the Arabian sea, include the coastal regions of the states of Maharashtra and Goa and span approximately 880 km. The climate of this region is humid tropical and it receives extensive rainfall during the monsoon season. Within the coastal zone there are a number of critical habitats, including estuaries, lagoons, mangroves, coral reefs, seagrass beds, tidal mudflats, backwaters, salt marshes, rocky coasts, sandy stretches and oceanic islands [45], which harbour a rich diversity of biota, including coral reefs [46] and mangrove forests [47]. However, studies on the microbial diversity of this region are scarce [8, 48].

In this study, we prepared an inventory of cultivable actinobacteria from sponges and associated environments of the intertidal zones along the northern parts of the western coast of India and studied their molecular diversity based on 16S rRNA gene sequences. We screened a subset of randomly selected cultures for predatory activity, antibiotic production and enzyme inhibition, and tested their associations with each other and with the isolation source to test the hypotheses mentioned earlier.

## Methods

### Sample collection

Small tissue samples (<1 g) of marine sponges (*Haliclona* spp.) were collected at low tide along the Maharashtrian and Goan coast (18–15°N and 73–74°E) of India during April 2014 to October 2018 without damaging the sponge or its associated environment. Specimens were rinsed and flushed with sterile distilled water using a dropper multiple times to remove debris and loosely attached microbes without damaging the tissue. Each sponge sample was collected in labelled polystyrene tubes with lids containing sterile poor Ravan saline (PRS) [49] and Zobell marine broth (ZMB) (Table 1)) [50]. Sediment, water and air samples were collected from the same environment as that of the sponge and were collectively considered as environmental samples. Sediment samples were collected directly inside sterile polypropylene tubes by immersing the tubes at the bottom of the rocky pool. Air samples were collected as a control to ensure that the isolates obtained from sponge, sediment or water were not air contaminants. For air sample collection, sterile polypropylene tubes containing sterile media were exposed to the air around the sampling spots. The samples were brought to the laboratory maintaining cold chain and were immediately processed for microbial culturing.

**Table 1.** Composition of poor Ravan saline (PRS) and Zobell marine broth (ZMB)

Medium	Ingredients	Concentration (g l^−1^)
Poor Ravan saline*	Glucose	0.050
	Peptone,	0.050
	Yeast extract	0.050
	Sodium acetate	0.050
	Sodium citrate	0.050
	Pyruvic acid	0.050
Zobell marine broth*†	Peptone	5.000
	Yeast extract	1.000
	Ferric citrate	0.100
	Sodium chloride	19.450
	Magnesium chloride	8.800
	Sodium sulphate	3.240
	Calcium chloride	1.800
	Potassium chloride	0.550
	Sodium bicarbonate	0.160
	Potassium bromide	0.080
	Strontium chloride	0.034
	Boric acid	0.022
	Sodium silicate	0.004
	Sodium fluorate	0.003
	Ammonium nitrate	0.002
	Disodium phosphate	0.008

*Solid media containing 15 g l^−1^ of agar.

†Final pH 7.6±0.2 at 25 °C.

### Isolation and maintenance of cultivable actinobacteria

Sediment samples were subjected to pre-heat treatment at 60 °C for 15 min to reduce the numbers of Gram-negative bacteria commonly found in marine samples, which often overcrowd the isolation plate [51]. Sediment samples vortexed in sterile saline were allowed to settle and supernatant was serially diluted up to 10^−5^. Samples (0.1 ml) were spread into triplicates on Petri plates containing sterile medium. Similarly, dilutions of the water samples were also plated. Sponge tissue (0.1 cm^3^) was homogenized in sterile saline and vortexed for 5 min. Tubes were left undisturbed for 2 min. From the resulting supernatant, serial 10-fold dilutions up to 10^−5^ were made and 0.1 ml samples were spread in triplicates on Petri plates containing sterile medium. We used two solid media, Zobell Marine Agar (ZMA) and poor Ravan saline agar (PRSA) (Table 1), with and without the antibiotic chloramphenicol (25 µg ml^−1^). Antibiotic producers are known to harbour the most antibiotic resistance genes [52]; therefore, use of a broad-spectrum antibiotic is expected to enhance selective isolation of antibiotic-rich organisms. Chloramphenicol was used as a broad-spectrum antibiotic for this purpose. Plates were incubated at 30 °C for 7 days in the case of ZMA and 21 days for PRSA. Plates were observed regularly for the growth of actinobacteria. Bacterial colonies that showed resemblance to actinobacteria under a light microscope were purified four–five times on respective media to ensure axenic culture. In all, 237 actinobacterial isolates were selected and were restreaked to make pure cultures. Colonies were labelled as per the Maharashtra Gene Bank (MGB) project code and preserved on ZMA slants at 4 °C for further use. Similarly, glycerol (18%) stocks were prepared and maintained at −20 °C for long-term storage. Actinobacterial cultures were deposited in the Microbial Culture Collection (MCC) of the National Centre for Microbial Resource, National Centre for Cell Sciences, Pune, India (accession numbers are provided in Table S1, available in the online version of this article).

### Genetic identification, phylogeny and species delimitation

Actinobacterial genomic DNA was extracted using the Wizard Genomic DNA Purification kit (Promega, USA) following the manufacturer’s protocols. The full-length (1.5 kb) 16S rRNA gene was amplified using polymerase chain reaction (PCR) with universal primers 27F (5′–AGA GTT TGA TCM TGG CTC AG–3′) and 1492R (5′–TAC GGY TAC CTT GTT ACG ACT T–3′) [53]. PCR was performed on the total reaction volume of 25 µl containing 5 µl template DNA (~200 ng), 2.5 µl of 10× reaction buffer (100 mM Tris pH 9.0, 500 mM KCl, 15 mM MgCl_2_, 0.1 % gelatin), 1 µl of 10 mM dNTPs, 1 µl of each 5 µM primer and 0.5 µl of 5 U µl^−1^ *Taq* polymerase and nuclease-free water to make up the final volume. The thermal profile was 2 min at 94 °C, and 25 cycles of 10 s at 98 °C, 30 s at 53 °C and 1 min at 68 °C, followed by an extension of 10 min at 68 °C. Amplified DNA fragments were purified using the Wizard Gel and PCR clean up (Promega, USA) system and sequenced bidirectionally using the BigDye Terminator v3.1 Cycle Sequencing kit (Applied Biosystems). Chromatograms of the sequences were manually checked for quality using BioEdit [54]. Forward and reverse sequences were assembled to form the complete 16S rRNA gene using GeneStudio sequence analysis software version 2.2.0.0 (GeneStudio, Suwanee, GA, USA; http://www.genestudio.com/).

The gene sequences used for the study were deposited in the GenBank database under the accession numbers MN339687–MN339897 and MT598037–MT598065 (Table S1). Sequences were checked in blast [55] to find the closest sequences available in the GenBank database (http://www.ncbi.nlm.nih.gov). Four species of Firmicutes, namely *Bacillus paralicheniformis* (MCC 6306), *Bacillus thuringiensis* (MCC 7835), *Bacillus subtilis* (MCC 6386) and *Bacillus halotolerans* (MCC 8381), were used as outgroups (GenBank accession numbers MN339894–MN339897, respectively).

Gene sequences were aligned using muscle [56] implemented in mega 7 [57]. The final aligned matrix had 1595 sites. The best nucleotide substitution model was determined using ModelFinder [58] based on the Bayesian information criterion [59, 60]. Maximum-likelihood analysis was performed in IQ-TREE [61] with ultrafast bootstrap support [62] for 1000 iterations. The phylogenetic tree was edited in FigTree v1.4.2 [63].

To understand the putative number of actinobacterial species we performed species delimitation based on Poisson tree processes [64] with maximum-likelihood partitioning (mPTP) and Bayesian partitioning (bPTP). A maximum-likelihood tree was used to delimit species by setting the parameter values as follows: MCMC generations=100 000, thinning=100, burn-in=0.1 and seed=123.

### Screening for activities

Out of 237 actinobacterial isolates, 50 isolates were selected randomly for the screening of 3 activities, namely predation, antibiotic production and production of enzyme inhibition.

### Target bacteria used for predation and antibiotic screening

Test bacteria of clinical importance, used for checking actinobacterial predation and antibiotic production, were obtained from MCC and National Collection of Industrial Microorganisms (NCIM), National Chemistry Laboratory, Pune, India. Fourteen bacteria, namely *Acetobacter pasterianus* (NCIM 2317), *Alcaligenes fecalis* (NCIM 2262), *B. subtilis* (NCIM 2063), *Enterococcus fecalis* (MCC 6462), *Escherichia coli* (NCIM 2184), *Klebsiella pneumonae* (NCIM 2957), *Micrococcus luteus* (NCIM 2673), *Mycobacterium smegmatis* (NCIM 5138), *Proteus vulgaris* (NCIM 2172), *Pseudominas aeruginosa* (NCIM 5029), *Salinicoccus roseus* (MCC 7574), *Salmonella enterica* (NCIM 2501), *Serretia marcescens* (NCIM 2919) and *Staphylococcus aureus* (NCIM 2121), were used as target species for screening.

### Screening for actinobacterial predatory growth

The growth of predators with the zone of clearance on prey cells was considered as predation as defined earlier [41]. The method for the preparation of prey cells was modified from Kumbhar *et al.* [41] and Pund *et al.* [42]. Pure cultures of the prey species were inoculated on nutrient agar plates to check the purity and were later reinoculated in nutrient broth. Inoculated flasks were incubated at 37 °C for 24 h. Broth culture was centrifuged at 6026 ***g*** for 10 min to concentrate cells using the Eppendorf centrifuge 5810R. Cells were washed thrice with sterile distilled water to remove traces of nutrient broth. Pellets were suspended in saline to obtain a thick suspension with an optical density of 1.0 at 600 nm. A lawn of prey cells (0.1 ml) was spread on water agarose plates and plates were incubated at 37 °C for 40 min. Fresh actinobacterial culture was spot inoculated on preincubated plates. These plates were incubated at for 48–72 h at 30 °C. Plates with plaques were examined visually and by using 4 and 45× magnification under a light microscope. Prey and predator control plates were used for comparison. Each experiment consisted of triplicate sets of plates, as well as one predator control for testing the growth of an actinobacterial predator without prey. In addition, there was a prey control to demonstrate viable and independent growth of prey without a predator. In either control there was no zone of clearance, indicating that there was no predation in the presence of predator or prey alone.

### Screening for antibacterial activity using the conventional cross-streak method

Selected actinobacterial cultures were screened for antibacterial activity using the cross-streak method [65, 66]. The test organism was streaked as a straight line along the diagonal of the Petri dish with sterile ZMA medium. The isolated pure colony of actinobacteria was inoculated as a single streak perpendicular to the central streak. Streaking was done from the edge of the plate to the test organism growth line. Plates were incubated at 37 °C for 18 h. The microbial inhibition was observed by determining the zone of clearance around the sensitive organisms. Inhibition activity was recorded qualitatively as presence or absence. Control plates of the same medium with the streak of test bacteria and without the streak of actinobacteria growth were used to observe the normal growth of the test bacteria.

### Screening for enzyme inhibitors

Actinobacterial cultures were screened for their ability to inhibit the activity of serine proteases and angiotensin-converting enzyme (ACE). Inhibition of serine proteases was selected to test our hypothesis that predatory actinobacteria will have efficient inhibitors of lytic enzymes, while ACE inhibition was selected because it has clinical applications. Three different serine proteases, i.e. subtilisin, trypsin and α-chymotrypsin, were used to screen for inhibitory activity. Protease inhibitor activity was studied using unprocessed X-ray films and the spot test method [67] with modifications. As described by Tripathi *et al.* [68], dilutions of pure enzyme were first spotted on gelatine-coated films. The lowest dilution showing complete clearance (indicating complete digestion of gelatine) was chosen for further studies. Pure enzyme (100 µg ml^−1^) was incubated with an equal volume of cell-free supernatant of actinobacterial isolates (10 µl each) for 10 min and transferred to untreated X-ray-Fuji Medical X-ray HRU grade films. The mixtures were allowed to react for 15 min at room temperature and the results were recorded after the X-ray films were washed under running water. Unprocessed X-ray films contain a layer of gelatine on their surface, which acts as a substrate for various proteolytic enzymes. Degradation of gelatine gives a clear zone at the site of activity. Thus, upon the action of the proteases, clear zones were seen on unprocessed X-ray films, at the site of inoculation, whereas, if the gelatine layer remained intact, no clearance was observed. No clearance on the films indicated the presence of protease inhibitors.

ACE acts on a specific substrate N-hyppuryl-His-Leu (HHL) to liberate hippuric acid and His-Leu. Liberated hippuric acid was detected spectrophotometrically. Upon reaction of the enzyme with ACE inhibitors, the enzyme becomes inactive and this is measured in terms of lower levels of hippuric acid being released. The protocol suggested by Cushman and Cheung [69] was used with certain modifications and hippuric acid liberation was checked using the method suggested by Ng *et al.* [70]. Equal volumes of ACE (100 µg ml^−1^) and cell-free supernatants (10 µl each) were allowed to react at 37 °C. After 10 min 20 µl of HHL was added to the reaction mixture and the reaction was continued for 30 min at 37 °C. The reaction was stopped by the addition of 40 µl of 1 N HCl. A blank was prepared by the addition of HCl before the addition of the substrate. A positive enzyme control was prepared by incubating enzyme with uninoculated broth. Liberated hippuric acid was extracted in 90 µl ethyl acetate by vigorous shaking. The ethyl acetate layer was collected in a fresh vial and allowed to dry in a water bath at 50 °C. The liberated hippuric acid was diluted in 150 µl distilled water and absorbance was checked at 228 nm. Zero was adjusted using distilled water. Test vials with more than 15 % inhibition of ACE were considered to be positive for ACE inhibitor.

### Statistical analysis

Association between the source of isolation and the frequency of occurrence of three activities (predation, antibiosis and enzyme inhibition) and association between the frequency of occurrence of different activities was tested using the χ^2^ test of association with the null hypothesis that there is no significant association. The null hypothesis that there was no significant difference in the frequency of actinobacterial isolated that showed antibiosis or predatory activity against Gram-negative and Gram-positive test organism was tested using the Mann–Whitney U test. All statistical tests were evaluated at α=0.05. Statistical tests were performed using the freeware PAST 4.0 [71].

## Results

### Actinobacterial phylogenetic diversity in sponge and associated environments

**Fig. 1.**
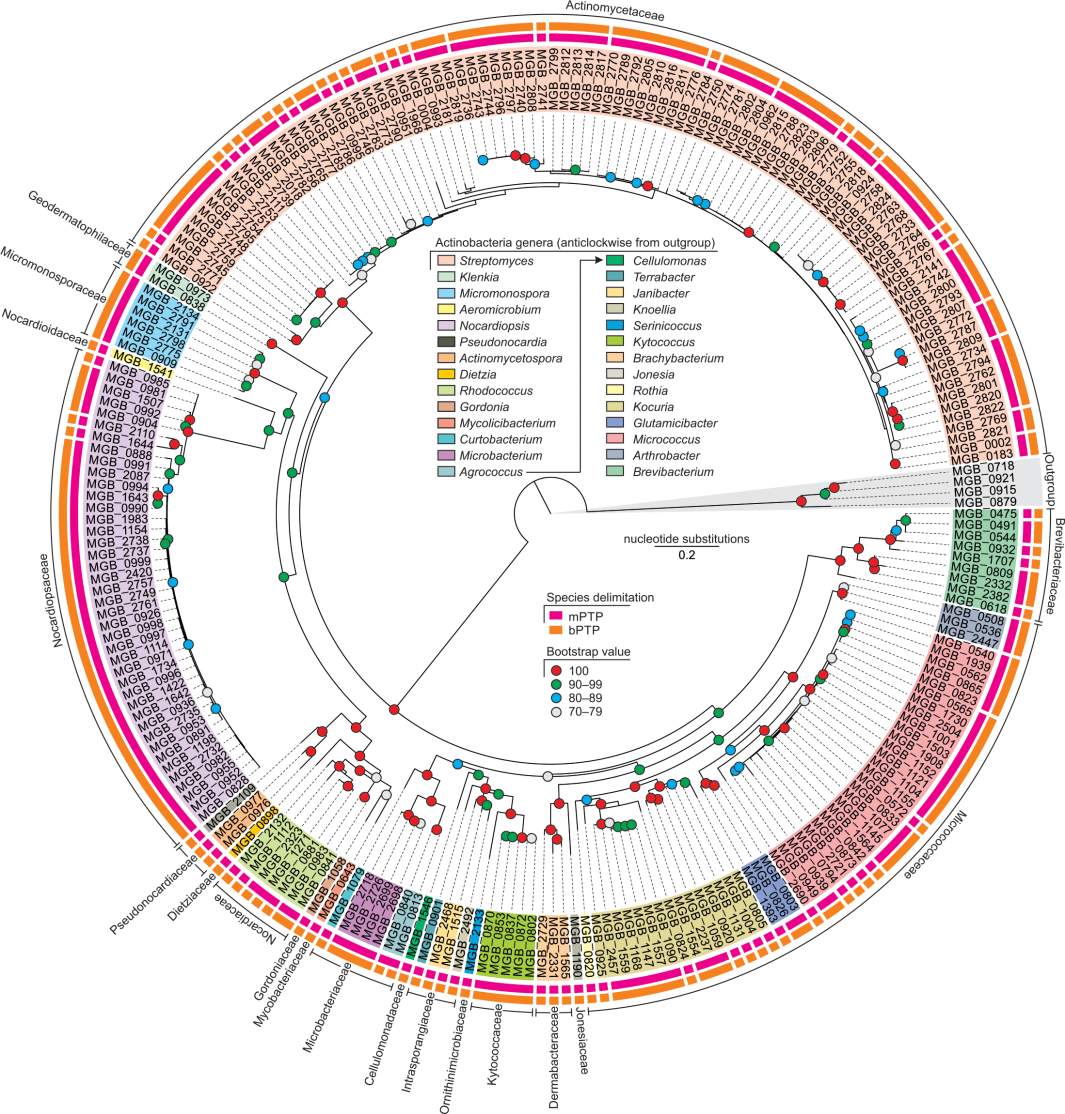
Maximum-likelihood phylogenetic tree of actinobacterial isolates based on the TIM3+F+I+G4 nucleotide substitution model (lnL of consensus tree: −18 684.58). Firmicutes belonging to genus *Bacillus* were used as outgroups.

The phylogenetic diversity of actinobacteria from sponges and associated environments is shown in Fig. 1. We obtained 237 actinobacterial isolates, from sponge and associated environments, belonging to 19 families and 28 genera (Table S1). Species delimitation based on mPTP suggested that these isolates belong to 95 putative species, while bPTP suggested 100 putative species. The two species delimitation methods, mPTP and bPTP, differed in the groups of species under the genera *Micrococcus *, *Rhodococcus* and *Streptomyces* (Table S1). We only recovered 3 isolates from air, belonging to the genera *Brachybacterium *, *Brevibacterium* and *Rhodococcus *, as compared to 39 isolates from water, 105 isolates from sediment and 90 isolates from sponge, suggesting that the isolates from water, sediment and sponge were not due to air contamination.

From sponges, there were 18 genera under 14 families belonging to 56 putative species (Table 2). From the sponge-associated environment, 22 genera under 15 families were recorded belonging to 64 putative species as per mPTP and 65 putative species as per bPTP. A total of 12 genera under 9 families and 28 putative species based on mPTP and 25 putative species based on bPTP were common to both sponges and their associated environments.

**Table 2.** Putative number of species of actinobacterial genera based on mPTP and bPTP methods isolated from sponge, associated environments and both sources

Family	Genus	Sponge		Environment		Both	
		mPTP	bPTP	mPTP	bPTP	mPTP	bPTP
Actinomycetaceae	* Streptomyces *	23	23	24	25	12	11
Brevibacteriaceae	* Brevibacterium *	5	5	2	2	1	1
Cellulomonadaceae	* Cellulomonas *	0	0	1	1	0	0
Dermabacteraceae	* Brachybacterium *	1	1	2	2	0	0
Dietziaceae	* Dietzia *	0	0	1	1	0	0
Geodermatophilaceae	* Klenkia *	1	1	1	1	1	1
Gordoniaceae	* Gordonia *	1	1	0	0	0	0
Intrasporangiaceae	* Janibacter *	0	0	2	2	0	0
	* Knoellia *	0	0	1	1	0	0
	* Terrabacter *	0	0	1	1	0	0
Jonesiaceae	* Jonesia *	1	1	0	0	0	0
Kytococcaceae	* Kytococcus *	0	0	1	1	0	0
Microbacteriaceae	* Agrococcus *	1	1	1	1	1	0
	* Curtobacterium *	0	0	1	1	0	0
	* Microbacterium *	1	1	1	1	1	1
Micrococcaceae	* Arthrobacter *	1	1	1	1	1	1
	* Glutamicibacter *	0	0	2	2	0	0
	* Kocuria *	4	4	6	6	2	2
	* Micrococcus *	6	6	8	8	4	4
	* Rothia *	1	1	0	0	0	0
Micromonosporaceae	* Micromonospora *	1	1	1	1	1	1
Mycobacteriaceae	* Mycolicibacterium *	1	1	0	0	0	0
Nocardiaceae	* Rhodococcus *	2	2	2	2	2	1
Nocardioidaceae	* Aeromicrobium *	0	0	1	1	0	0
Nocardiopsaceae	* Nocardiopsis *	4	4	3	3	2	2
Ornithinimicrobiaceae	* Serinicoccus *	1	1	0	0	0	0
Pseudonocardiaceae	* Actinomycetospora *	0	0	1	1	0	0
	* Pseudonocardia *	1	1	0	0	0	0
	Total	56	56	64	65	28	25

Six genera, namely *Gordonia *, *Jonesia *, *Mycolicibacterium *, *Pseudonocardia *, *Rothia* and *Serinicoccus *, were isolated only from sponges (Tables 2 and S1), which could be identified as species *Gordonia terrae* (MCC 6452), *Jonesia denitrificans* (MCC 7852), *Mycolicibacterium poriferae* (MCC 6242), *Pseudonocardia kongjuensis* (MCC 7930) and *Rothia terrae* (MCC 7823) and *Serinicoccus marinus* (MCC 7935), respectively. Although 12 genera, namely *Agrococcus *, *Arthrobacter *, *Brachybacterium *, *Brevibacterium *, *Klenkia *, *Kocuria *, *Microbacterium *, *Micrococcus *, *Micromonospora *, *Nocardiopsis *, *Rhodococcus* and *Streptomyces *, were isolated from both sponges and associated habitats, most of these genera had some putative species that were exclusive to either sponges or associated environments. In particular, seven species, *Brachybacterium muris* (MCC 7614), *Brevibacterium casei* (MCC 6140, MCC 6152, MCC 6176), *Kocuria rhizophila* (MCC 8384), *Nocardiopsis salina* (MCC 7931), *Rhodococcus zopfii* (MCC 7934), *Streptomyces smyrnaeus* (MCC 7924) and *Streptomyces viridobrunneus* (MCC 7990), were recorded only from sponges.

With respect to both the number of isolates and the number of putative species, *Streptomyces* was the most dominant genus, and was found in both sponges and associated environments. *Nocardiopsis* was the second most common genus, with two dominant species *Nocardiopsis alba* (MCC 8385) followed by *Nocardiopsis dassonvillei* (MCC 7845). Among the genera and species that were only recorded from the environment, we provide the first record of species such as *Aeromicrobium massiliense* (MCC 6739) and *Glutamicibacter mysorens* (MCC 7825) from marine waters.

### Non-obligate epibiotic predatory activity

**Fig. 2.**
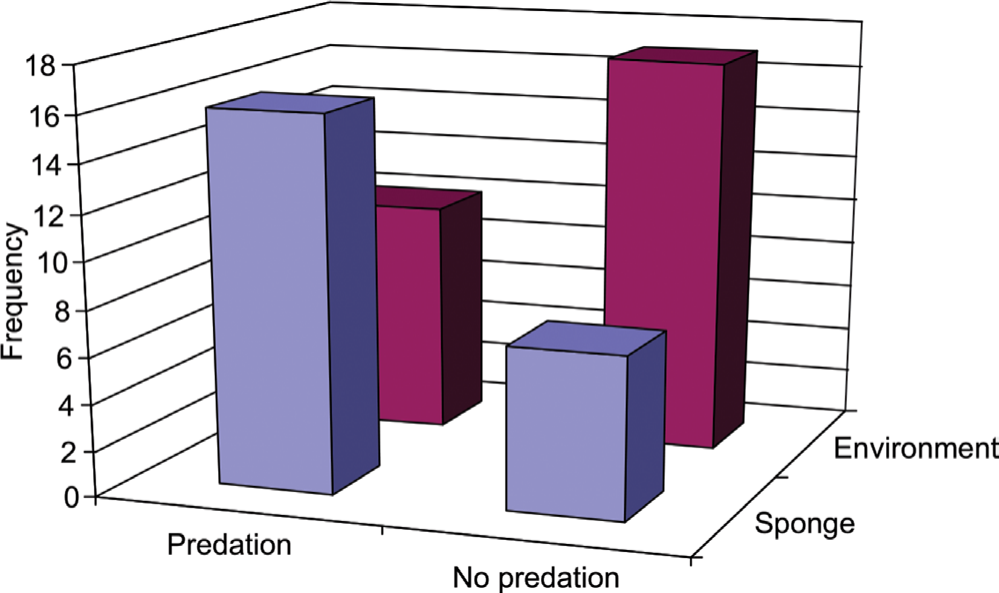
Association between source of actinobacterial isolation and predatory behaviour. There was a significant association between the source (sponge or associated environment) of actinobacterial isolation and predation (χ^2^=5.265, *P*=0.0218).

Out of the total of 50 actinobacterial isolates screened for non-obligate epibiotic predatory activity, 26 isolates showed predation against at least 1 of the 14 target organisms (Table S2). Of the 26 isolates with predatory behaviour, 17 preyed on Gram-negative prey, 21 preyed on Gram-positive prey, and 12 preyed on both Gram-negative and Gram-positive prey. There was no significant difference (Mann–Whitney U=15, *P*=0.2601) in the frequency of actinobacterial predators on Gram-negative and Gram-positive prey (Table 3). Most actinobacterial predators (*n*=14) preyed on a single prey species, while only a few predators preyed on multiple prey species. A single predator of the genus *Streptomyces* preyed on eight prey species. There was a significant association between the source of isolation (sponge or associated environment) and predatory behaviour (χ^2^=5265, *P*=0.0218), where the isolates from sponge showed proportionately more predatory behaviour (Fig. 2).

**Table 3.** Predation and antibiotic production by actinobacteria against the Gram-positive and Gram-negative target species

Target species	Predation	Antibiotic	Predation and antibiotic by same actinobacterial isolate
Gram-positive			
* Mycobacterium smegmatis *	3	12	0
* Micrococcus luteus *	8	5	0
* Bacillus subtilis *	1	24	1
* Staphylococcus aureus *	17	9	4
* Salinicoccus roseus *	9	3	0
* Enterococcus faecalis *	3	20	1
Gram-negative			
*Acetobacter pasterianus*	7	0	0
* Alcaligenes faecalis *	3	1	1
* Escherichia coli *	2	5	0
* Klebsiella pneumoniae *	3	0	0
* Proteus vulgaris *	8	0	0
* Salmonella enterica *	2	0	0
* Serratia marcescens *	3	0	0
* Pseudomonas aeruginosa *	1	0	0

All eight isolates of *Streptomyces* used for screening showed predatory behaviour and preyed on both Gram-negative and Gram-positive prey (Table S2). Out of 25 isolates of *Nocardiopsis *, 12 showed predatory behaviour, out of which 5 preyed on Gram-negative bacteria while 11 preyed on Gram-positive bacteria. Both *Micromonospora* isolates preyed on Gram-positive prey, while only one preyed on Gram-negative prey. Isolates belonging to the genera *Brevibacterium *, *Glutamicibacter* and *Rhodococcus* only preyed on Gram-negative prey, while *Rothia* only preyed on Gram-positive prey.

### Antibiosis, antibacterial activity and growth inhibition

Of the 50 actinobacterial isolates screened for antibacterial activity, 25 showed antibiosis against at least 1 target organism (Table S2). Of these 25 isolates, all showed antibiosis against at least 1 of the Gram-positive target species, while only 5 showed antibiosis against at least 1 of the Gram-negative organisms. The frequency of antibacterial activity against Gram-positive organisms was significantly higher (Mann–Whitney U=1.5, *P*=0.003) than against Gram-negative organisms (Table 3). Most antibacterial activities were broad spectrum with respect to the target organisms that they affected. There were 10 actinobacterial isolates that showed antibiosis against 2 target organisms, 6 isolates that affected 4 target species and 2 isolates that affected 6 target species. There was no association between antibacterial activity and the source (sponge or associated environment) of the isolation (χ^2^=2.0129, *P*=0.1560).

Out of eight *Streptomyces* isolates that were screened for antibacterial activity, five showed antibiosis, of which two showed antibiosis against Gram-negative target species, while all showed antibiosis against Gram-positive organisms. In the case of *Nocardiopsis *, of the 25 isolates used for screening, 17 showed antibiosis, of which all affected the growth of Gram-positive organisms, while only 2 affected the growth of Gram-negative organisms. Genus *Kytococcus* showed antibiosis that affected both Gram-positive and Gram-negative organisms, while *Glutamicibacter* and *Rothia* only showed antibiosis against Gram-positive organisms.

### Enzyme inhibition

**Fig. 3.**
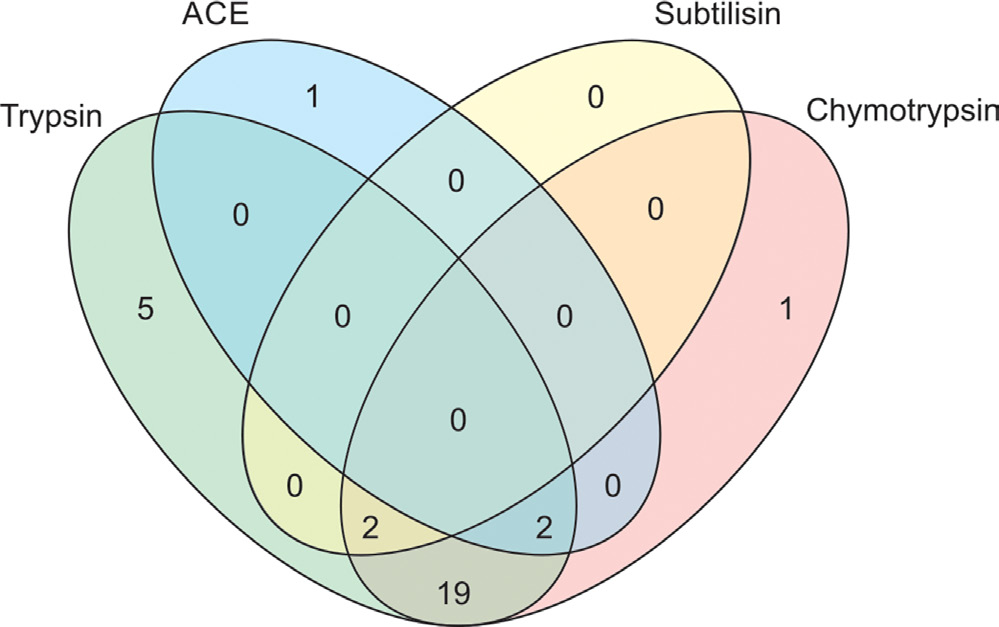
Venn diagram combination of enzyme inhibitors produced by actinobacterial isolates. Venn diagram is not to scale.

Out of 50 actinobacterial isolates screened for the inhibition of 4 enzymes, 30 isolates inhibited at least 1 of the enzymes (Table S2). Of these 30 isolates, 28 inhibited trypsin, 24 inhibited chymotrypsin, 3 inhibited angiotensin-converting enzyme (ACE) and only 2 inhibited subtilisin. A Venn diagram of the frequency of isolates inhibiting different enzymes (Fig. 3) suggested that five isolates only inhibited trypsin and one isolate each inhibited chymotrypsin and ACE, while subtilisin inhibition was accompanied by the inhibition of other enzymes. No isolate inhibited all four enzymes. Out of 30 actinobacteria that produced enzyme inhibitors, 19 produced 2 inhibitors, 4 produced 3 inhibitors and 7 produced only one of the 4 inhibitors. There was no association between enzyme inhibition and the source of the actinobacterial isolate (χ^2^=2.3386, *P*=0.1262).

Out of 8 isolates of *Streptomyces *, 7 produced enzyme inhibitors against proteases, while 12 out of 25 isolates of *Nocardiopsis* produced enzyme inhibitors, of which 11 worked against proteases and 2 worked against ACE (Table 4). One isolate of *Actinomycetospora* inhibited the activity of ACE.

**Table 4.** Frequency of actinobacterial isolates producing four different enzyme inhibitors

Genus	No. of isolates	Frequency of isolates inhibiting				Isolates with at least one inhibition activity
		Subtilisin	Trypsin	Chymotrypsin	ACE	
* Actinomycetospora *	2	0	1	0	1	2
* Agrococcus *	1	0	0	0	0	0
* Brevibacterium *	1	0	1	1	0	1
* Glutamicibacter *	1	0	1	1	0	1
* Jonesia *	1	0	0	0	0	0
* Kocuria *	1	0	0	0	0	0
* Kytococcus *	1	0	1	0	0	1
* Micrococcus *	1	0	1	0	0	1
* Micromonospora *	2	0	2	2	0	2
* Nocardiopsis *	25	0	11	11	2	12
* Pseudonocardia *	1	0	0	0	0	0
* Rhodococcus *	4	0	2	1	0	2
* Rothia *	1	0	1	1	0	1
* Streptomyces *	8	2	7	7	0	7

### Associations between different activities

**Fig. 4.**
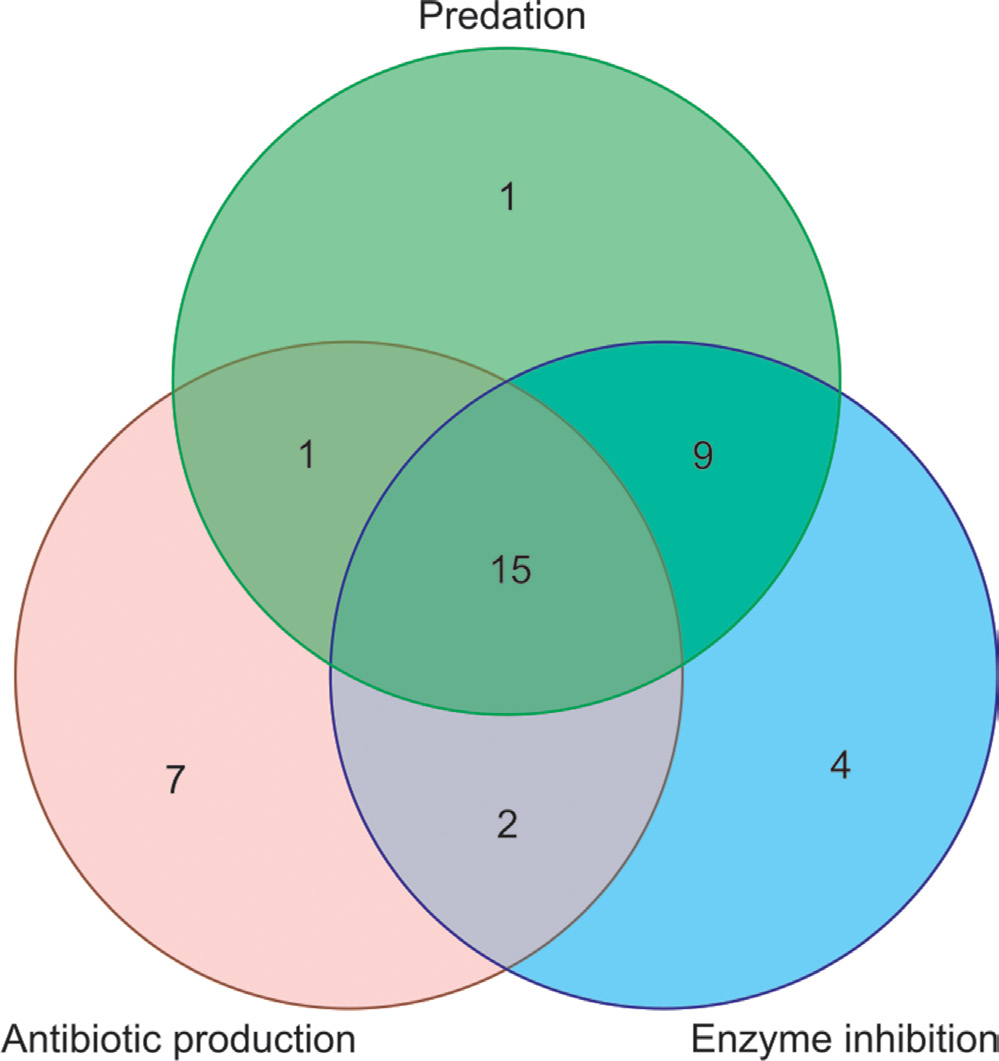
Venn diagram of predation, antibiotic production and enzyme inhibition by actinobacterial isolates. Venn diagram is not to scale.

Out of 50 actinobacterial isolates that were screened for activities, 39 showed at least 1 of the 3 activities. Of these 39 isolates, 15 showed all 3 activities, while 9 showed predation as well as enzyme inhibition (Fig. 4). There were only seven isolates that showed predation and antibiotic production against the same target organism (Table 3) and all of these isolates belonged to the genera *Streptomyces* and *Nocardiopsis *.

**Fig. 5.**
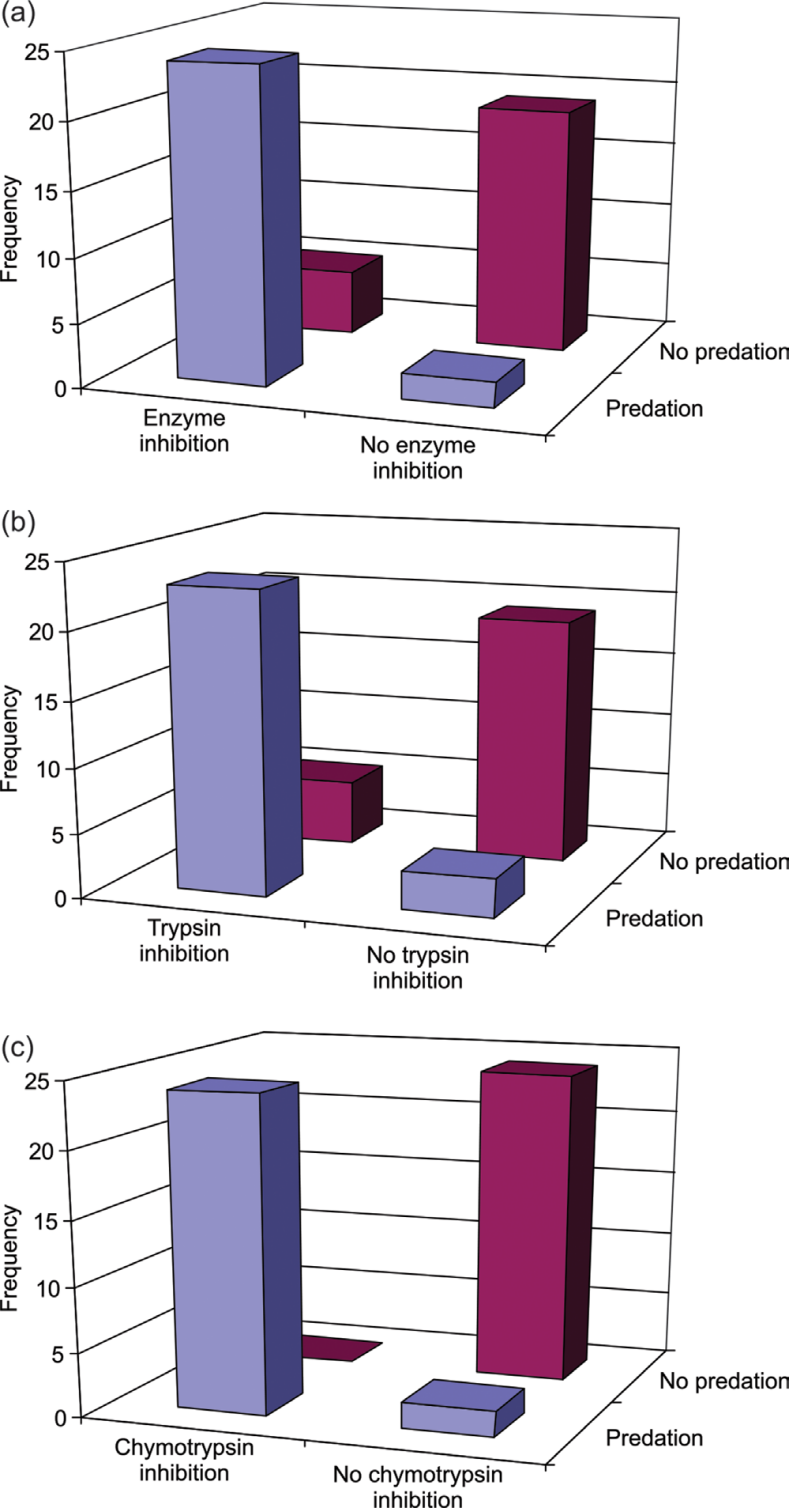
Association between enzyme inhibition and predation in actinobacterial isolates. Predation was significantly associated with (**a**) inhibition of any one of the four enzymes tested (χ^2^=26.172, *P*<0.0001), (**b**) inhibition of trypsin (χ^2^=23.165, *P*<0.0001) and (**c**) inhibition of chymotrypsin (χ^2^=42.604, *P*<0.0001).

Antibiotic production showed no significant association with predation (χ^2^=2.8846, *P*=0.0894) or inhibition of any of the four enzymes (χ^2^=2.0525, *P*=0.1520). However, there were significant associations between predation and protease inhibitors (Fig. 5). Twenty-four isolates showed both predation and inhibition of at least one enzyme, and there was a significant association between the two activities (χ^2^=26.172, *P*<0.0001), where predators produced proportionally more enzyme inhibitors than non-predators (Fig. 5a). Twenty-three actinobacterial isolates showed both predation and trypsin inhibition, and there was a significant association between the two (χ^2^=23.165, *P*<0.0001), with predators more likely to produce trypsin inhibitors than non-predators (Fig. 5b). Similarly, 24 actinobacteria were predatorsand inhibited chymotrypsin activity, and there was a significant association between the two (χ^2^=42.604, *P*<0.0001), with predators more likely to produce chymotrypsin inhibitors than non-predators (Fig. 5c).

## Discussion

Sponges and associated environments in the northern parts of the western coast of India are rich in actinobacterial diversity, with ~95 putative species under 19 families and 28 genera. We recorded 13 species of actinobacteria solely from sponges. Of these, *Mycobacterium poriferae* was originally described from marine sponge [72], while three species, *Gordonia terrae* [21, 73, 74], *Brevibacterium casei* [75] and *Kocuria rhizophila* [76], have previously been reported from sponges. To our knowledge, we provide the first report of nine species, namely *Brachybacterium murisi, Jonesia denitrificans*, *Nocardiopsis salina *, *Pseudonocardia kongjuensis *, *Rhodococcus zopfii *, *Rothia terrae *, *Serinicoccus marinus *, *Streptomyces smyrnaeus* and *Streptomyces viridobrunneus *, from marine sponges, although some of them are known from marine habitats [77–80].

*Streptomyces* was the most dominant genus among the isolates, which agrees with the findings of Zhang *et al.* [81]. Genus *Nocardiposis*, with its two species *Nocardiposis alba* and *Nocardiposis dassonvillei*, has been suggested [82] as the second most common genus after *Streptomyces* and that too agrees with our findings. Further, the reporting of the most prevalent genera, including *Agrococcus *, *Arthrobacter *, *Brevibacterium *, *Kocuria *, *Microbacterium* and *Micrococcus *, from sponges in our study is consistent with previous reports from other study areas, including the South China Sea [17], the Yellow Sea [81], the Mediterranean Sea [26], the coast of Florida in the USA [21] and the northern coast of Brazil [83], indicating that there are common trends in the discovery of actinobacteria from sponges.

Among the first reports from the marine environment from our study, *Aeromicrobium massiliense* and *Glutamicibacter mysorens* are known from human faecal microbiota [84] and sewage [85], respectively. The presence of these two species in the sediments along the collection site Harne (17.81°N, 73.09°E) may suggest faecal pollution in this area. Runoff from nearby urban, rural and agricultural lands could also explain the presence of other known terrestrial actinobacterial species from marine environments.

Although predation is a widespread behaviour in the bacterial kingdom, δ-proteobacteria of the orders *Myxococcales* and *Bdellovibrionales* have received more attention [86] than other taxa, especially Gram-positive bacteria such as actinobacteria. Among the phylum Actinobacteria, only three genera, namely *Agromyces *, *Streptomyces* and *Streptoverticillium *, are known to have epibiotic predatory behaviour against other bacterial species [39–42, 87–89]. In the current study, for the first time, we show predation in six other genera of the phylum Actinobacteria, namely *Brevibacterium *, *Glutamicibacter *, *Micromonospora *, *Nocardiopsis *, *Rhodococcus* and *Rothia *. Kumbhar *et al.* [41] argued that predatory behaviour is widespread in the genus *Streptomyces* and even in the current study we observed that all the isolates of *Streptomyces* used for screening showed predation on Gram-positive as well as Gram-negative prey.

Since sponges are sessile and lack other anti-predator defences, it has been suggested that secondary metabolites of bacteria can provide sponges with chemical defence [34, 38]. However, we did not observe any significant association between the source of actinobacterial isolation and antibiotic production, suggesting that isolates even from environment were equally likely to produce antimicrobials as isolates recovered from sponges. Our finding therefore supports the recent suggestions that antibiotics may have other roles, including as intermicrobial signalling agents, instead of just being weapons [34, 90]. Nevertheless, there was a significant association between the source of isolation and predatory activity, with proportionately more predators among the isolates recovered from sponge. Ecologically this makes sense. As the sponges are filter feeders and have regular intake of environmental bacteria, sponge-associated actinobacteria will have better predation opportunities. It is also possible that the predatory activity of sponge-associated actinobacteria could have evolved as a mutualistic activity, as it can defend sponges from pathogenic bacterial invasions.

Actinobacteria are known to produce several enzyme inhibitors [29, 30, 91]. However, for the first time we show a strong association between predation and enzyme inhibition, specifically inhibition of trypsin and chymotrypsin, where predators produced proportionally more enzyme inhibitors than non-predators. Predators themselves are known to produce a variety of hydrolytic enzymes for degrading prey [92]. Therefore, it is possible that the production of enzyme inhibitors safeguards their own cells from being a target of the enzyme. It is also possible that enzyme inhibitors also protect actinobacteria from hydrolytic enzymes produced from the sponge host and other microbiota.

An interesting observation that we made, when comparing predation and antibiotic production by actinobacteria, was that, while predation was equally effective against Gram-positive and Gram-negative target species, antibiotic production was mainly effective against Gram-positive bacteria. Recently, Ibrahimi *et al.* [89] suggested that there are some bio-active secondary metabolites that co-cultured actinobacteria produce in the presence of prey cells. It is therefore possible that studying the predatory behaviour of actinobacteria and predation-specific metabolites could lead to the discovery of novel therapeutic agents that are more broad spectrum.

Although actinobacteria are known to be rich in secondary metabolites, extracellular enzymes and enzyme inhibitors, the ecological role of these extracellular bioactive molecules is little known. We suggest that studying the ecological correlates of bioactivity and the inter-correlation patterns of different types of bioactivity can be a useful tool in understanding the ecological origins of bioactivity and testing alternative ecological hypotheses.

## Conclusion

Sponges and their associated environments in intertidal zones along the northern parts of the western coast of India are rich in actinobacterial diversity, with 19 families and 28 genera, which could be attributed to 95 putative species using mPTP and 100 putative species based on bPTP methods. Although at the genus level the trends in the discovery of actinobacteria isolated from sponges were consistent with previous studies from different study areas, we provide the first report of nine species, namely *Brachybacterium murisi, Jonesia denitrificans*, *Nocardiopsis salina *, *Pseudonocardia kongjuensis *, *Rhodococcus zopfii *, *Rothia terrae *, *Serinicoccus marinus *, *Streptomyces smyrnaeus* and *Streptomyces viridobrunneus *. Non-obligate epibiotic predatory behaviour was widespread among actinobacterial genera and we provide the first report of predatory activity in *Brevibacterium *, *Glutamicibacter *, *Micromonospora *, *Nocardiopsis *, *Rhodococcus* and *Rothia *. Sponge-associated actinobacteria showed significantly more predatory behaviour than environmental isolates, and we hypothesize that predatory actinobacteria might provide sponges with defence against pathogenic bacteria. While antibiotics produced from actinobacterial isolates affected Gram-positive target bacteria, with little to no effect on Gram-negative bacteria, predation targeted both Gram-positive and Gram-negative prey with equal propensity, suggesting that study of predation specific metabolites might provide novel therapeutic agents with broad-spectrum activity. Actinobacterial isolates from both sponge and associated environments produced inhibitors of serine proteases and angiotensin-converting enzyme. Predatory behaviour was strongly associated with inhibition of trypsin and chymotrypsin, which might be helpful for the actinobacteria in overcoming the effects of proteolytic enzymes produced by sponge host and other microbiota. Understanding the diversity and associations among various actinobacterial activities – with each other and the source of isolation – can provide new insights into marine microbial ecology and provide opportunities to isolate novel therapeutic agents.

## Data availability

The sequences of the 16S rRNA gene of the studied isolates were submitted to the National Center for Biotechnology Information’s (NCBI’s) GenBank under the accession numbers MN339687–MN339897 and MT598037–MT598065. Actinobacterial cultures were deposited in the Microbial Culture Collection (MCC) of the National Centre for Microbial Resource, National Centre for Cell Sciences, Pune, India (accession numbers are provided in the Table S1). All the data used for analysis are provided in Tables S1 and S2.

## Supplementary Data

Supplementary material 1
